# Scrotal abscesses in perianal Crohn’s disease: a retrospective cohort study of clinical presentation, management, and outcomes

**DOI:** 10.1007/s10151-026-03319-3

**Published:** 2026-05-12

**Authors:** F. Dermuth, M. Oruc, M. DeWitt-Foy, M. Fascelli, H. Kessler, S. D. Holubar

**Affiliations:** 1 Dept. of Colorectal Surgery, Digestive Disease Institute, Cleveland Clinic, 9500 Euclid Avenue A30, Cleveland, OH 44195 USA; 2 Dept. of Urology, Cleveland Clinic, Cleveland, OH USA

**Keywords:** Fistulizing perianal Crohn’s disease, Perianal abscess, Scrotal abscess

## Abstract

**Background:**

Perianal Crohn’s disease (pCD) is a severe phenotype frequently complicated by fistulas and recurrent abscesses. Although perianal sepsis is well described, genital involvement is rare, with scrotal abscesses previously reported only in case reports. Consequently, their presentation, management, and outcomes remain poorly understood.

**Methods:**

We conducted a retrospective cohort study of male patients with Crohn’s who developed scrotal abscesses between January 1, 2009, and October 31, 2024, at our quaternary referral center. Clinical records were reviewed for demographics, disease phenotype, abscess characteristics, diagnostic modality, management, microbiology, and clinical outcomes.

**Results:**

A total of 40 patients were identified. Median age at abscess presentation was 42 years (IQR 34.5–50), and median BMI was 24.7 kg/m^2^ (IQR 22.3–31.4). Most patients had colonic disease (57.5%) and long-standing Crohn’s disease. Prior Crohn’s-related abscesses occurred in 82.5%, and 37.5% had a history of perianal seton placement. Abscesses were most commonly right-sided (57.5%), and 35% of patients had a scrotal fistula. Diagnosis was most frequently established using computed tomography (32.5%). All patients received antibiotics; 30% underwent bedside incision and drainage, and 37.5% underwent operative drainage. Hospital admission was required in 60%, recurrence within 12 months occurred in 40%, and escalation of advanced therapy in 20%. During follow-up, 35% required fecal diversion (72% or 25% overall), and 20% underwent proctectomy.

**Conclusion:**

Scrotal abscesses are an uncommon but clinically significant complication of pCD, occurring in patients with severe perianal disease and are associated with substantial morbidity. Early recognition and multidisciplinary management are essential for successful outcomes.

## Introduction

Perianal Crohn’s disease (pCD) is a severe manifestation of Crohn’s disease (CD) that presents with perianal lesions, such as perianal fistulas, abscesses, anal skin tags, strictures, and ulcers. Overall, up to 25% of patients with CD have perianal disease, and approximately 11.5% of patients with pCD present with perianal disease at the time of initial CD diagnosis [1, 2]. The risk of developing perianal disease within 10 years after CD diagnosis is reported to be between 18.9% [2] and 29.5% [3].

Perianal CD is a more aggressive phenotype with suboptimal long-term outcomes including more interventions, imaging, hospitalizations and higher economic burden compared to the non-perianal phenotypes [4, 5]. In addition to the increased risk of major abdominal surgery, and the need for a fecal diversion with a stoma, patients with pCD are increased risk of malignant transformation of the fistulae into anorectal adenocarcinoma [6]. Furthermore, quality of life (QoL) is negatively impacted by this disease phenotype due to chronic pain, psychological stress, and chronic drainage caused by the perianal lesions, resulting in a high physical and psychological burden for the patients [7].

Crohn’s disease is characterized by transmural inflammation of the bowel wall, which predisposes patients to complications such as fistula formation and abscess development. In penetrating CD, particularly when the distal colon or perianal region is involved, the close anatomic proximity of pelvic structures may allow disease-related infection to extend into adjacent genitourinary organs, resulting in urinary, genital, and reproductive organ complications [8, 9]. The incidence of urinary complications in patients with CD has been reported to be approximately 7% [10], whereas genital and reproductive organ involvement remains poorly characterized due to limited available data. These complications in both women and men are associated with a high disease burden, often due to abdominopelvic pain and discharge, and carry an increased risk of pelvic abscesses and sepsis if not adequately controlled [8]. In female patients, disease extension may manifest as rectovaginal fistulas, vulvar involvement, or, in rare cases, enterosalpingeal fistulas [8, 11, 12].

In male patients, genitourinary and reproductive organ complications are most commonly associated with perianal disease and may present as urethral, vesical, or penile fistulas, prostatic abscesses, or penile and scrotal lymphedema [8, 13]. Among the spectrum of genitourinary manifestations in male patients with pCD, scrotal involvement represents a particularly rare and understudied complication. Scrotal abscesses in this setting remain poorly characterized, with existing evidence largely limited to isolated case reports and case series [14, 15]. A clearer understanding of this rare complication may facilitate its early recognition, enable timely management, and support better-informed treatment decisions. Early recognition is particularly important given the involvement of male reproductive organs and the predominance of young male patients in the pCD population.

This study aimed to describe the clinical presentation, management, and outcomes of scrotal abscesses in patients with pCD. In addition, we aimed to compare different initial management strategies and assess the associated recurrence rates. To our knowledge, this study represents the largest cohort to date that examines this uncommon yet clinically significant complication in a complex patient population.

## Materials and methods

### Study design, data collection, and outcomes

After obtaining approval from the institutional review board, we conducted a retrospective cohort study using a prospectively maintained institutional database. Male patients with pCD who were diagnosed with scrotal abscesses between January 1, 2009, and October 31, 2024, were identified and retrospectively analyzed. A comprehensive chart review was performed for all eligible patients. Patients with scrotal abscesses arising from causes unrelated to CD, such as trauma, postoperative complications, or primary urologic infections were excluded.

Collected variables included baseline demographic data (e.g., age, body mass index, American Society of Anesthesiologists (ASA) classification, and surgical history), CD characteristics including current medical therapy, scrotal abscess features (e.g., symptoms, laterality, and diagnostic modality), management strategy (antibiotic therapy alone, bedside incision and drainage, or incision and drainage in the operating room), and clinical outcomes, including 6-month and 12-month recurrence and escalation of inflammatory bowel disease (IBD) medical therapy.

“Overall resolution” of the abscess was defined as clinical and, when available, radiological resolution, including absence of abscess on imaging, resolution of local inflammatory signs, absence of symptoms, and complete wound healing without persistent discharge following incision and drainage.

The primary objective of this study was to describe the clinical presentation, microbiology, management, and outcomes of scrotal abscesses as a rare complication of pCD. The secondary objective was to compare the treatment approaches and their associated recurrence rates.

### Statistical analysis

Descriptive analyses were performed to characterize patient demographics and clinical features. Continuous variables were reported as medians with interquartile ranges (IQRs). Categorical variables were summarized as frequencies and proportions. Differences between groups were assessed using the Mann–Whitney* U* test for continuous variables and Fisher’s exact test for categorical variables. A* p* value of less than 0.05 was considered statistically significant. Statistical analyses were performed using R software (R Foundation for Statistical Computing, Vienna, Austria).

## Results

### Baseline patient and Crohn’s disease characteristics

A total of 40 (100%) patients were identified and included in the study (Table 1). The median age at abscess presentation was 42 years (IQR 34.5–50), and the median BMI was 24.7 kg/m^2^ (IQR 22.3–31.4). Of the patients, 52.5% were active or former smokers. According to the Montreal classification, age at CD onset was most commonly A2 (17–39 years) in 18 patients (45%), followed by A3 (≥ 40 years) in 10 patients (25%), and A1 (≤ 16 years) in 7 patients (17.5%). Colonic disease (L2) was present in 23 patients (57.5%), and 15 patients (37.5%) had ileocolonic disease. The median time between CD diagnosis and scrotal abscess diagnosis was 12 years (IQR 5–21.5). At the time of scrotal abscess diagnosis, 14 patients (35%) were receiving biologic therapy, 8 (20%) immunomodulators, 7 (17.5%) corticosteroids, 6 (15%) 5-aminosalicylates (5-ASA), and 1 patient (2.5%) small-molecule targeted therapy. While 7 patients (17.5%) received more than one treatment, 15 patients (37.5%) were not receiving IBD-specific medical therapy.

**Table 1:** Baseline demographics and Crohn’s disease phenotype in patients with scrotal abscesses (*n* = 40)

Variable	*n* (%)
Age at diagnosis	42 (34.5–50)
BMI, kg/m^2^	24.7 (22.3–31.4)
Smoking	
Active	12 (30)
Former	9 (22.5)
Never	19 (47.5)
Montreal classification	
Age at diagnosis	
A1, ≤ 16 years	7 (17.5)
A2, 17–39 years	18 (45)
A3, ≥ 40 years	10 (25)
Unknown	5 (12.5)
Behavior	
L2, colonic	23 (57.5)
L3, ileocolonic	15 (37.5)
Unknown	1 (2.5)
CD duration until abscess diagnosis, years	12 (5–21.5)
IBD therapy at diagnosis*	25 (62.5)
Biologics	14 (35)
Immunomodulator	8 (20)
Corticosteroids	7 (17.5)
5-ASA	6 (15)
Small-molecule targeted therapy	1 (2.5)
More than one treatment	7 (17.5)
No treatment	15 (37.5)

Figures represent median (IQR) or frequency (proportion)

*BMI* body mass index,* CD* Crohn’s disease,* IBD* inflammatory bowel disease

*Some patients received more than one treatment agent

A history of CD-related abscesses with associated surgery was present in 33 patients (82.5%). Perianal setons had been previously placed in 15 patients (37.5%), with 6 patients (15%) having setons in situ at the time of scrotal abscess diagnosis.

### Scrotal abscess presentation, diagnosis, and treatment

**Fig. 1:**
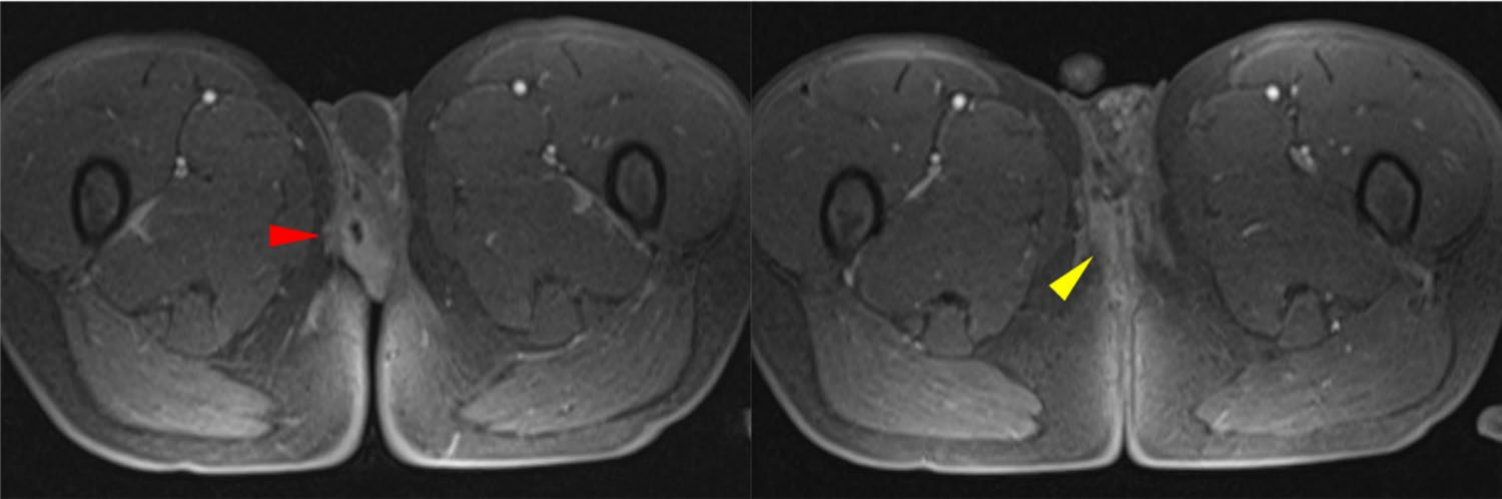
Axial MRI showing right scrotal abscess (red arrow) with a perianal fistulous tract extending to the scrotum (yellow arrow). © Cleveland Clinic Foundation 2026

**Fig. 2:**
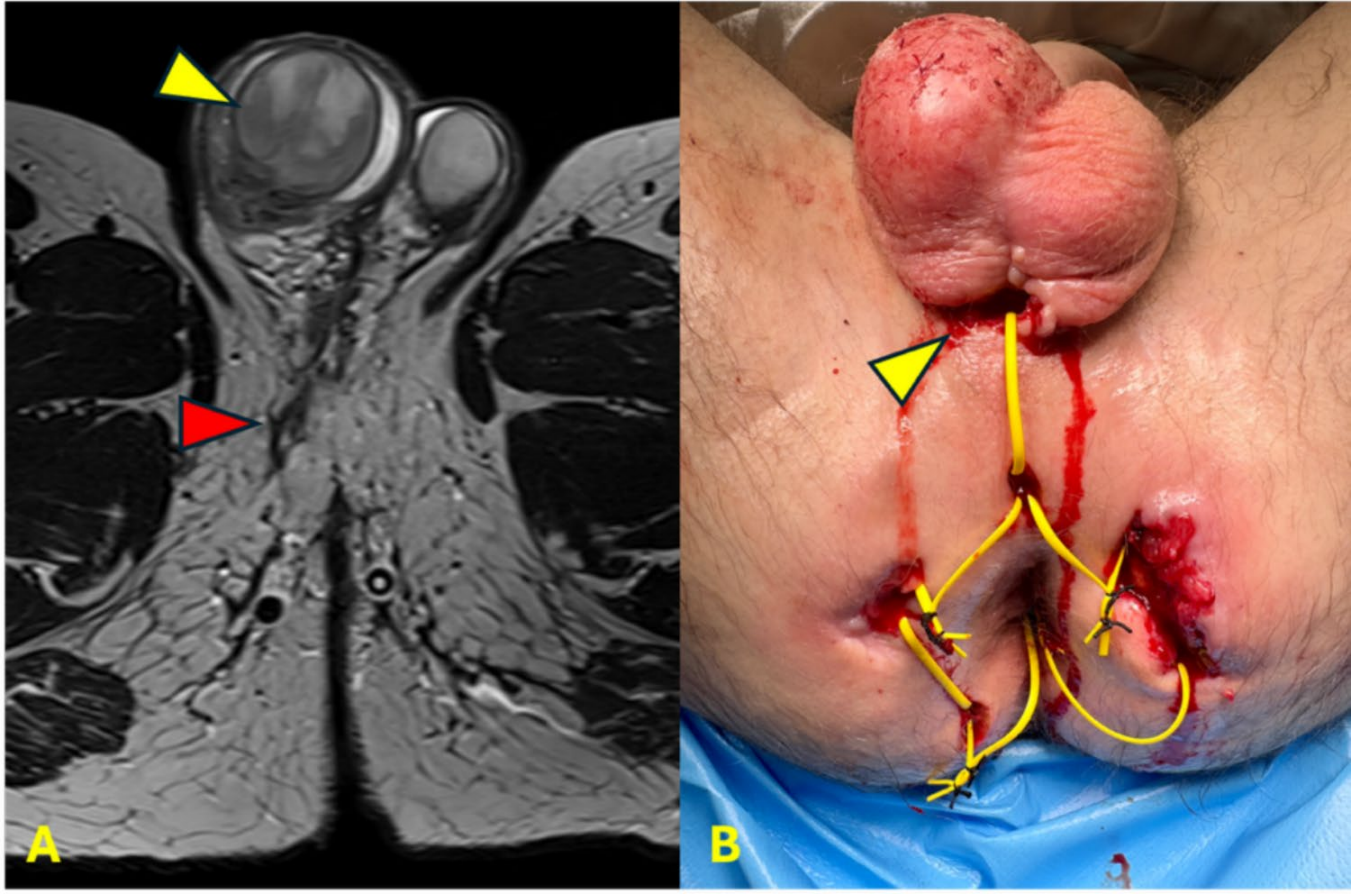
MRI revealed scrotal and testicular abscesses (yellow arrow) and fistulous tracts reaching to the base of the scrotum (red arrow) (**A**). Surgical rationalization with placement of a total of six setons for drainage of the fistulous network, one reaching the base of the scrotum (yellow arrow) (**B**). © Cleveland Clinic Foundation, 2026

Scrotal abscesses were most commonly right-sided (57.5%), followed by left-sided (25%), midline (10%), and bilateral involvement (7.5%). Scrotal discharge was reported in 15 patients (37.5%), and a scrotal fistula was identified in 14 (35%, Figs. 1 and 2). Concomitant testicular involvement in the form of a testicular abscess was observed in one patient (2.5%). Symptoms at initial presentation included pain and swelling (100%), fever (42.5%), and some patients also reported diarrhea (12.5%) or bloody diarrhea (5%). Diagnosis was most frequently established using computed tomography (CT) in 13 patients (32.5%), followed by ultrasound in 11 patients (27.5%), clinical diagnosis alone in 9 patients (22.5%), and magnetic resonance imaging (MRI) in 7 patients (17.5%, Figs. 1 and 2).

All patients received antibiotic therapy, with systemic antibiotics administered to all hospitalized patients (Table 2). Thirteen patients (32.5%) were managed with antibiotics alone, 12 (30%) underwent bedside incision and drainage, and 15 (37.5%) were treated with incision and drainage under general anesthesia in the operating room. In five patients (12.5%), incision and drainage was combined with concomitant seton placement during the index procedure. In two of those patients, definitive seton removal was achieved during follow-up. Microbiological cultures were obtained from 11 patients (27.5%), with 8 patients (20%) yielding positive results. *Escherichia coli* was the most frequently isolated organism. At discharge (either hospital or emergency department discharge), amoxicillin/clavulanate was the most commonly prescribed antibiotic (40%), followed by sulfamethoxazole/trimethoprim and ciprofloxacin (each 17.5%). Other agents included cephalexin and tinidazole (each 12.5%), with less frequent use of doxycycline, levofloxacin, and vancomycin. Some patients received combination antibiotic therapy.

**Table 2:** Antibiotic treatment upon discharge in patients with Crohn’s disease and scrotal abscesses (*n* = 40)

Variable	*n* (%)
Amoxicillin/clavulanate	16 (40%)
Sulfamethoxazole/trimethoprim	7 (17.5%)
Ciprofloxacin	7 (17.5%)
Cephalexin	5 (12.5%)
Tinidazole	5 (12.5%)
Doxycycline	3 (7.5%)
Tinidazole	2 (5%)
Levofloxacin	2 (5%)
Vancomycin	1 (2.5%)

Figures represent frequency (proportion)

*Some patients received more than one treatment agent

### Clinical outcomes

Overall resolution of the scrotal abscess following initial management occurred in 26 patients (65%). No statistically significant differences in abscess resolution were observed between the treatment groups (*p* = 0.68, Table 3). Hospital admission was required in 24 patients (60%), with a median length of stay of 8 days (IQR 4–9 days). Within 30 days of discharge, 8 patients (20%) required hospital readmission. Recurrence of scrotal abscesses within 12 months occurred in 16 patients (40%), with no statistically significant differences between the management strategies (*p* = 0.69). Following abscess management, escalation of IBD-specific medical therapy was necessary in 8 patients (20%).

**Table 3:** Abscess resolution and 12-month recurrence by initial treatment modality in patients with Crohn’s disease and scrotal abscesses (*n* = 40)

	Antibiotics only (*n* = 13)	Bedside I&D (*n* = 12)	OR I&D (*n* = 15)	*p* value
Resolution of abscess				
Yes	8 (20%)	7 (17.5%)	11 (27.5%)	0.68
No	5 (12.5%)	5 (12.5%)	4 (10%)	
12-month recurrence				
Yes	4 (10%)	5 (12.5%)	7 (17.5%)	0.68
No	9 (22.5%)	7 (17.5%)	8 (20%)	

Figures represent median (IQR) or frequency (proportion)

*I&D* incision and drainage,* OR* operating room

### Subsequent surgical interventions

During follow-up after scrotal abscess diagnosis, a subset of patients required additional surgical interventions for perianal Crohn’s disease (Table 4). The median follow-up duration was 5.9 years (IQR 2.4–9.9).

**Table 4:** Further surgical interventions after initial abscess drainage in patients with Crohn’s disease and scrotal abscesses (*n* = 40)

Variable	*n* (%) or median (IQR)
Fecal diversion	19 (37.5%)
Preexisting	5 (12.5%)
Subsequent	14 (35%)
Loop ileostomy	10 (25%)
End ileostomy	4 (10%)
Temporary	4 (10%)
Permanent	10 (25%)
Time to diversion, months	21 (2–41)
Proctectomy	14 (35%)
Preexisting	6 (15%)
Subsequent	8 (20%)
Time to proctectomy, months	12 (6–23)
Orchiectomy*	1 (2.5%)
EUAs	1 (0–2.25)
Intervention under anesthesia^†^	1 (0–4%)

Figures represent frequency (proportion) or median (IQR)

*EUA* exam under anesthesia

*Orchiectomy was performed due to testicular involvement

^†^Interventions under anesthesia includes incisions, drainages, debridement, and fistulotomy

Additional examinations under anesthesia (EUAs) were required in 25 patients (62.5%), with a median of one EUA per patient (IQR 0–2). Subsequent perianal surgical interventions, including repeat drainage procedures or fistula-related surgery, were performed in 26 patients (65%), with a median of one intervention per patient (IQR 0–4).

Fecal diversion was performed in 14 patients (35%), with a median time to diversion of 21 months (IQR 2–41) following scrotal abscess diagnosis. Among these patients, 4 diversions (28%) were temporary, and 10 (72%, or 25% overall) were permanent. Five patients (12.5%) had already undergone fecal diversion prior to scrotal abscess diagnosis. Proctectomy was ultimately required in 8 patients (20%), performed at a median of 12 months (IQR 6–23) after abscess presentation. Indications for proctectomy included medically refractory fistulizing perianal Crohn’s disease in five patients and progressive abscess formation with recurrent pelvic sepsis in three patients. Orchiectomy was performed in one patient (2.5%) due to concurrent testicular abscess with an unsalvageable testis.

## Discussion

This study provides the largest contemporary description of scrotal abscesses as a rare complication of perianal Crohn’s disease and highlights their association with severe disease and adverse clinical outcomes.

Genitourinary and reproductive organ complications are rare but recognized and clinically important manifestations in pCD. To date, no incidence data have been reported in adult patients, while the incidence of genitourinary complications in pediatric patients has been reported to be 9% [16]. Data on scrotal or testicular abscesses are scarce and limited to case reports, and no outcome-focused cohort has been described to date [14, 15, 17].

The incidence of abscess formation in perianal fistulizing CD is known to be high, with reported rates of up to 66% [18]. This is accompanied by a substantial need for perianal interventions and seton placement in patients. In our cohort, 82.5% of the patients had a history of perianal abscesses before scrotal abscess diagnosis, and 37.5% had undergone prior seton placement. These findings suggest that scrotal abscesses occur in a subset of patients with perianal Crohn’s disease characterized by a high disease burden. Fistulous extension to the scrotum has not been well defined and was identified in 35% of our cohort, suggesting that the presence of a scrotal fistula should raise awareness of this potential subsequent complication.

In our cohort, 37% of patients were not receiving active medical therapy for Crohn’s disease at the time of abscess diagnosis. The reasons for absence of treatment likely include prior discontinuation of therapy or other patient-related factors. Although insufficient disease control may theoretically contribute to abscess development, current evidence does not clearly establish a direct causal relationship between treatment status and abscess presentation. Instead, abscess formation appears to be more closely associated with underlying disease phenotype and inflammatory activity. This heterogeneity should be considered when interpreting our findings [19].

The recommended diagnostic modalities for abscesses and fistulas in pCD include contrast-enhanced magnetic resonance imaging (MRI) and computed tomography (CT). However, in emergency settings, CT is often the preferred modality owing to its wider availability [20]. This is reflected in our data, with 32.5% of patients being diagnosed using CT. In the acute setting of perianal sepsis, it is particularly important to exclude Fournier gangrene, for which contrast-enhanced CT is the preferred imaging modality [21].

The management of scrotal abscesses in perianal Crohn’s disease is heterogeneous and frequently multimodal. We observed no significant differences in abscess resolution or recurrence between the initial management strategies, suggesting that outcomes are more strongly influenced by the severity of the underlying perianal disease rather than the specific intervention employed, consistent with prior observations in CD-associated perianal sepsis [22, 23]. Although orchiectomy was required in only one patient, its occurrence underscores the potential severity of scrotal involvement and potential testicular involvement in perianal Crohn’s disease and the risk of irreversible reproductive organ damage in advanced cases.

More invasive management options for severe perianal Crohn’s disease include fecal diversion and proctectomy. Fecal diversion may be beneficial in patients with perianal sepsis to promote local disease control and healing; however, the proportion of patients who ultimately undergo restoration of bowel continuity remains low, with reported rates of 10–17% [24–26]. Proctectomy rates in perianal Crohn’s disease have been reported to range between 10% and 20%, with studies demonstrating improvement in quality of life following definitive surgery [27]. 

Scrotal abscesses recurred frequently in our cohort, underscoring the relapsing nature of perianal Crohn’s disease. Reported recurrence rates of perianal abscesses following surgical treatment range from approximately 54% at 2 years to 62% at 5 years, while lower recurrence rates have been described in patients receiving optimized medical disease management [28, 29]. In this context, the observed 40% 1-year recurrence rate of scrotal abscess as a rare genital complication in our cohort is notable and highlights the aggressive and chronic disease course in patients who develop scrotal involvement.

Rather than indicating failure of initial abscess management, these recurrences are more likely attributable to ongoing inflammatory activity and persistent fistulizing disease in patients with advanced perianal Crohn’s disease.

Management of scrotal abscesses in patients with perianal Crohn’s disease required interdisciplinary collaboration. Depending on clinical presentation, care involved several specialties, including colorectal surgery, general surgery—often the first team evaluating patients in the emergency department—urology, radiology, and gastroenterology. In particular, gastroenterology was consulted for optimization of medical therapy and overall treatment planning. Multidisciplinary management of complex Crohn’s disease has been associated with favorable short-term outcomes, supporting the importance of coordinated care in this challenging patient population [30].

## Conclusion

Scrotal abscesses represent a rare but clinically significant manifestation of perianal Crohn’s disease, with a high rate of recurrence, admission, readmission, and need for subsequent surgical interventions. Improved characterization facilitates earlier recognition, supports timely and multidisciplinary management, and enables more informed treatment decisions. Early recognition is particularly important given the involvement of male reproductive organs and the young age of many patients with perianal Crohn’s disease.

## Data Availability

The data used in this study are not publicly available but can be provided upon reasonable request.

## Declarations

### Prior publication

This manuscript is original and has not been previously submitted or published elsewhere.
